# Whole-body Magnetic Resonance Imaging in the German National Cohort (NAKO): Design & Current Status

**DOI:** 10.1093/eurpub/ckac129.029

**Published:** 2022-10-25

**Authors:** F Bamberg

**Affiliations:** Department of Radiology, University Hospital Freiburg, Freiburg, Germany

## Abstract

**Background:**

Whole-body magnetic resonance imaging (MRI) permits non-invasive, non-ionizing phenotyping of the human body and ideally complements the epidemiological assessment of the NAKO participants. As such, it allows for the detection of morphologic or functional predisposition, early disease stages prior to overt clinical events as well as evident pathological changes. The assessment of progression and regression of imaging phenotypes over time will provide the basis to identify and understand the relevance of imaging-based risk factor profiles for disease development.

**Methods:**

Integrated in the general NAKO study program and managed by a central Imaging Core, study participants underwent whole-body imaging at five dedicated MR imaging sites. Imaging was performed on five identical 3 Tesla scanners (Magnetom Skyra, Siemens Healthineers, Erlangen, Germany) applying a one hour protocol, including sequences for the brain, the cardiovascular and musculoskeletal system as well as for the thorax and abdomen. Comprehensive measures to assure high image quality and management of incidental findings were established.

**Results:**

As part of the baseline examination program, a total of 30,861 participants successfully underwent the MR imaging program. All measures of quality assurance and incidental findings management were successfully employed throughout the study period and obtained image quality and completeness of all MR sequences was excellent (>94.2% completeness). While MR imaging as part of the first re-examination is ongoing, baseline MRI data is currently accessible for scientific analyses.

**Conclusions:**

The MRI-Study of the NAKO will provide a comprehensive imaging phenotypic biobank covering different organ systems with highest morphological and functional detail. MRI data analysis will gain novel insights into the natural history of disease development, the role of subclinical disease burden, and revolutionize our understanding of imaging biomarkers of risk.

